# The Peregrinating Psychiatric Patient in the Emergency Department

**DOI:** 10.5811/westjem.2016.6.30179

**Published:** 2016-07-19

**Authors:** Scott A. Simpson, Jagoda Pasic

**Affiliations:** * University of Colorado, Denver, School of Medicine, Department of Psychiatry, Aurora, Colorado; † Denver Health Medical Center, Department of Psychiatry, Denver, Colorado; ‡ University of Washington School of Medicine, Department of Psychiatry, Seattle, Washington

## Abstract

Many emergency department (ED) psychiatric patients present after traveling. Although such travel, or peregrination, has long been associated with factitious disorder, other diagnoses are more common among travelers, including psychotic disorders, personality disorders, and substance abuse. Travelers’ intense psychopathology, disrupted social networks, lack of collateral informants, and unawareness of local resources complicate treatment. These patients can consume disproportionate time and resources from emergency providers. We review the literature on the emergency psychiatric treatment of peregrinating patients and use case examples to illustrate common presentations and treatment strategies. Difficulties in studying this population and suggestions for future research are discussed.

## INTRODUCTION

Patients who travel long distances and present for psychiatric care are familiar to emergency providers. Yet the epidemiology, diagnoses, and treatment needs of these “travelers” remain largely unknown. The ready accessibility of emergency department (ED) care compared to outpatient or inpatient mental health services is attractive to traveling patients who are unfamiliar with local healthcare providers or have few local social supports and no healthcare providers.^1^ This paucity of local resources complicates ED providers’ treatment and disposition planning. The act of traveling itself may occur as the result of mental illness or introduce stressors that exacerbate psychopathology.

Travel by psychiatric patients was first recognized as a complication of Munchausen’s syndrome.^2^,^3^ In Munchausen’s, or factitious disorder, patients present with the unconscious production of somatic symptoms and are prone to traveling long distances to seek care from new providers. In this context, peregrination – derived from the Latin *peregrinating*, “to travel abroad” – was used to describe a patient’s traveling. However, Munchausen’s is quite rare (<1% of consultation-liaison consults) relative to the observed frequency of long-distance patients presenting for emergency mental healthcare (6.6% in one study).^4^,^5^ Thus, factitious disorder is not likely the most common diagnosis among most peregrinating psychiatric patients.

This paper reviews the literature on patients who present for emergency psychiatric care after traveling long distances. The diagnostic and treatment considerations raised through a systematic literature review are framed with case examples. The authors then share strategies for managing peregrinating psychiatric patients based on the literature and their experience as medical directors of psychiatric emergency services at large public safety-net hospitals.

## METHODS

We reviewed the scientific literature for articles describing the prevalence, pathology, or treatment of patients who present to the ED after traveling. A search using the unrestricted terms “travel” and “psychiatry” was conducted in the PubMed and PsycINFO databases. No date limiters were used. Papers’ titles and abstracts were screened for a full-text review. PubMed’s “Related Articles” feature and a review of included papers’ bibliographies provided additional records. Figure demonstrates a flow chart of the article selection process.^6^

### Overview of the Literature

We identified 21 articles as potentially relevant to the treatment of traveling psychiatric patients in the ED. The most relevant articles included case series (two articles),^7^,^8^ case reports (three articles),^9^–^11^ and a review of pertinent pathology (one article).^3^ Four papers compared traveling ED patients to non-traveling controls.^5^,^12^–^14^ Another 12 papers addressed psychiatric sequelae of acute travel, including seven articles on jet lag. Because these patients may present to EDs for care, these articles were included in this review. Articles were almost entirely limited to descriptions of traveling patients or relevant pathology: Only one article focused on treatment recommendations – to help patients with psychotic disorders prevent decompensation due to jet lag – but was focused on outpatient, preventative treatment.^15^

### Identifying Travelers

Terminology and case definitions of patients who travel vary greatly. One study of ED patients used the term “long-distance patients,” defined as those arriving to the ED from greater than 100 miles away.^5^ Investigators in Hawaii studied travelers who had arrived to the state in the preceding four months^7^ or changed at least two time zones in the preceding 10 days.^12^ An Israeli study compared tourists traveling three or fewer time zones to those traveling seven or more.^16^

Other available definitions are not specifically used for emergency care. For example, an examination of psychiatric re-admission rates among inpatients with schizophrenia in Taiwan compared patients from “remote” and “non-remote” towns using a government definition accounting for “location, geography, and medical availability.”^17^ Government definitions of mobility not specific to clinical care include persons moving greater than 500 miles or across county or state lines.^18^,^19^

Travel and residential moves are common in American life, but it is unclear how these migration patterns apply to healthcare settings.^18^ In census data, very few Americans cite health reasons as the purpose of their move. Seriously mentally ill patients who lack residences are not readily captured or easily represented by census data.^20^ Similarly, persons not staying in paid accommodations are unlikely to be tallied by travel associations. Such homeless persons are exactly those frequently seen in psychiatric emergency services. And while health reasons are rarely a reason for moving or traveling, patients who do travel for healthcare might be expected to be sicker and use greater healthcare resources.

### Prevalence of Travelers in the ED

Only one study describes the prevalence of patients who travel long distances for emergency psychiatric care. In that cohort study of traveling patients, 6.6% of patients in a psychiatric emergency service lived more than 100 miles from the hospital’s city.^5^ Because the hospital was located in a rural area, the authors suspected a higher prevalence may exist among urban hospitals.

### Presentations of the Traveling Psychiatric Patient

The following brief cases illustrate ED presentations commonly described in the literature or treated by the authors. In studies that have compared travelers to non-travelers, patients appear more likely to have depressive, manic, or psychotic episodes.^12^,^16^ Patients coming to the ED from farther away within a metropolitan area may be relatively more likely to have severe mental illness and involuntary presentations.^13^,^14^

### Case 1

A 30-year-old man presents with suicidal ideation after relapsing on alcohol. He recently moved to the area after a long period of unemployment, anticipating work with an oil drilling company. However, the job fell through, he has no local friends or family, and he lacks money to return home. This anxiety and new homelessness triggered his relapse.

Persons who move to a new area to seek employment, new residential amenities, or for family/relationship reasons may present to EDs in crisis. These patients are unable to access their usual coping skills and social supports owing geographic isolation. Loneliness, frustration, or regret may be prominent; patients may present with depression and perhaps after a suicide attempt.^7^ In general, residential mobility is associated with greater neuroticism and lower conscientiousness.^19^ Because neuroticism is associated with adverse health outcomes at a population level, it may be that persons who move are more likely to require psychiatry and medical care.^21^

Tourists and short-term residents often present with acute anxiety reactions, often precipitated by a minor event in the context of separation from social supports, fatigue from prolonged travel, and jet lag:

### Case 2

A 40-year-old man presents with chest pain and shortness of breath. The emergency physician diagnoses a panic attack. The patient has been in town caring for his terminally ill mother, which has proven a financial burden and emotionally exhausting. Today, the patient became overwhelmed with anxiety that his wife and children may be ill as well. The patient has history of an anxiety disorder but recently ran out of medications after missing a follow-up appointment in his hometown.

These patients may present to the ED with panic attacks, somatic reactions, seemingly paranoid thoughts (e.g., “something terrible is happening to my family”), or disorganization and psychotic reactions.^8^,^22^ Anxiety reactions may be more common among patients traveling for funerals or illnesses in the family.^12^

Patients with mental illness are prone to decompensation while traveling. For example, patients with a history of depression or mania are at elevated risk for depression or mania when travelling across time zones.^15^,^16^,^23^ Travel disrupts medication adherence and limits access to a patient’s mental health professionals.^12^ More serious medical complications are possible: One report describes two patients with schizophrenia who developed possible neuroleptic malignant syndrome upon traveling to a warm tropical country.^24^

### Case 3

A 45-year-old woman comes to the ED from a rural part of the state. She has borderline personality disorder and a history of over a dozen overdoses. She recently lost her girlfriend, which prompted an overdose and inpatient admission near her home. However, she had a conflict with her inpatient team, left, and now arrives to your academic medical center “for a second opinion” to help her “stabilize on medications.”

This case exemplifies the presentation of an “intentional” traveler who travels consciously and purposefully to obtain care at a specific facility.^5^ In most ways, intentional travelers are not demographically or diagnostically different than local patients. In the study defining intentional patients, these patients appeared to travel to access the resources of a larger hospital or avoid the stigma of psychiatric treatment in their home communities.^21^ Some patients indulge in a “rescue fantasy” that they have traveled to a hospital that will help them; this fantasy can complicate discharge or disposition planning. Personality disorders are common among intentional patients.

As opposed to these intentional patients, “incidental” travelers do not specifically seek psychiatric care but nonetheless come to the ED’s attention. This group often includes patients who are hitchhiking or homeless and brought in by police or ambulance. These patients are significantly more likely than locals to have had prior psychiatric treatment, suffer psychotic and substance use disorders, and require inpatient hospitalization.^5^ Case four describes such a patient:

### Case 4

A 25-year-old man with a history of schizophrenia calls 911 fr*om a homeless shelter because of intense command auditory hallucinations to hurt himself. He has been in town for six days from out of state. He tells you he moved “because I needed to” and that he often remains in a city for about a month. He will leave town “when the voices tell me to.”*

Patients with schizophrenia and delusional disorder often present after traveling from another state with vague intentions or paranoia; for example, they may “want a change” or to “see the mountains.” Their knowledge of local resources is often similarly unclear, and they may be uncertain where to obtain shelter, food, or medication. Some patients present after calling 911 with suicidal ideation. An assessment quickly reveals that the patient uses this strategy repeatedly in new cities to obtain treatment and orient to local resources. These patients may become high utilizers over a short period before moving on to another city.

Patients with bipolar disorder are also prone to peregrination during manic episodes. These patients are unlikely to seek out care once in a new city. More likely, they present via police or ambulance after causing a disruption in the community. Co-occurring substance use is not uncommon.

Patients with personality, dissociative, and factitious disorders often arrive to emergency care in dramatic fashion:

### Case 5

*A 53-year-old man presents to the ED with memory loss. He had flagged down a bus driver seeking help. He describes having recently arrived to the city but cannot remember where he came from or even who he is. He cannot recall who his family members are and lacks any identifying papers (but has $600 in his sock).*^25^

### Case 6

A 30-year-old woman presents to the ED with chest pain and shortness of breath. She states she has protein C deficiency and a history of pulmonary emboli. New to town, she describes prior surgeries – she has a prominent midline abdominal incision – for “life-threatening” medical issues without manifest anxiety. She is talkative and cooperative with care but vague as to prior interventions and treating physicians.

Despite their intact thought process, these patients describe profound hallucinations, delusions, or cognitive symptoms that belie their ability to travel long distances. Case 6 characterizes classic Munchausen’s, in which patients peregrinate; change providers; seek recurrent medical interventions; and disclose dramatic but vague histories of present illness.^3^ (This patient may have true protein C deficiency as well.) Patients with dissociative fugue present complaining of amnesia or confusion.^9^,^10^ Elucidating the patient’s history through a collateral informant, clinicians typically identify a profound social stressor precipitating migration. Gander syndrome, another dissociative disorder, may be present among travelers and is characterized by *vorbeireden*, in which approximate answers are given to questions, but those answers suggest the patient has a sense of the correct answer.^11^ (E.g., How many legs does a dog have? *Five.*)

Malingering should be considered in patients who are uncooperative, demand controlled substances, display atypical or inconsistent symptoms, and have evident secondary gain.^26^ Malingering is seen among patients with antisocial personality disorder who travel to avoid legal prosecution or violent reprisals.

### Case 7

A 30-year-old woman is brought in by ambulance after being found unconscious at a bus station. She awakes with the administration of intravenous naloxone. She reports that she is traveling cross-country and used heroin during an overnight layover. Disinterested in treatment, she desires discharge to catch her bus.

Substance use is present in most psychiatric emergencies and prevalent among travelers.^27^ Vacationers, particularly students on spring break, are prone to excessive alcohol use.^28^ Some patients miss bus or airplane connections because they have been using substances; patients may then be approached by security services or self-present seeking detoxification in the ED. If stranded and unable to continue home, their distress only increases. The psychotic symptoms of schizophrenia or bipolar disorder may be exacerbated by stimulant, hallucinogen, or marijuana use. The availability of drugs may itself promote travel: the legalization of marijuana in Colorado and Washington states has prompted migration of homeless persons to those states.^29^,^30^

The act of travel itself may induce biological stress that induces psychiatric illness. Air travel has been associated with cognitive impairment, delirium, and psychosis among elderly patients.^31^,^32^ These acute complications likely arise from fatigue as well as disruptions in circadian rhythms and diurnal cortisol regulation.^16^,^33^

### Approach to Treating Travelers

Ill, isolated, and in need of treatment, the traveling patient in the ED presents unique challenges for treatment. Our review revealed no treatment recommendations for the treatment of traveling patients in the ED. We provide some guidance based on our experience in emergency psychiatry.

### Solicit a history

An evaluation of patients who travel must begin with a history of present illness that includes for how long the patient has been in town and why the patient moved. The timing of peregrination provides clues to changes in functioning, symptom severity, and reasons for presentation. This line of questioning naturally leads to an assessment of the patient’s self-care: Where is the patient staying? What are sources of food, clothing, and income? Do they have a cell phone to facilitate follow up? Table 1 lists several key questions for evaluation.

Understanding the patient’s travel pattern is important for treatment planning in the ED. How long does the patient typically stay in a city? How did the patient pay for travel previously? Patients who move every few weeks are unlikely to engage in case management. The need for inpatient hospitalization must be determined based on acute safety concerns, but we find that many traveling patients do not participate in subsequent follow-up care. Thus, for chronic travelers, inpatient hospitalization is typically unhelpful for improving prognosis or engagement in treatment.

### Assess familiarity with local resources

Clinicians then identify the patient’s local resources: What family or friends live in the area? What employment opportunities exist for the patient? Patients new to town often require orientation to local healthcare and shelter resources. Oftentimes, patient express suicidal ideation that may be ameliorated by a brief introduction to the city, meal sites, day shelters, and crisis services. A map that locates these services and provides phone numbers is invaluable.

For patients without a local social network, the clinician should learn where the patient’s supportive persons reside and how to connect the patient with them.

### Obtain collateral information

Obtaining prior medical records or identifying collateral informants for travelers can be difficult. Patients may be resistant to disclosure or unaware of where they received treatment. In these cases, several strategies are helpful. Insurance companies often have on-call clinicians who will review claims, share diagnoses, and even provide treatment notes regardless of geographic location; a patient disclosure authorization is not required to talk to the insurer. Chronically mentally ill patients are often treated by community mental health providers that closely collaborate with local crisis lines. We have found that calling the regional crisis line in the patient’s prior residence can elucidate contact information for a recent case manager or treatment records; at least, the crisis counselor can provide clues as to local hospitals and agencies that are likely to have treated the patient. Major city hospitals and mental health agencies in the traveler’s city of origin may be found through an Internet search or an informal survey of colleagues in the ED. Many peregrinating patients present to EDs with discharge paperwork from another hospital; these records are vital. Multi-state record systems such as ED information exchanges or prescription drug monitoring databases can report where patients have previously received care.

Contact information for family or friends may be identified through outside hospital records, among the patient’s belongings, or in the patient’s cell phone. These persons may offer information helpful for an evaluation as well as provide the patient a social network to assist in disposition or other problem-solving.

### Focus on safety and the acute presentation

Like any patient presenting to the ED, travelers merit treatment of acute pathology and a comprehensive assessment of suicide and violence risk. As with local patients, long-distance patients may benefit from pharmacotherapy for agitation, psychosis, or anxiety; detoxification and substance treatment services should be considered.

Specific interventions and disposition planning should reflect the patient’s local resources, individual strengths, and personal goals. Brief psychotherapy for traveling patients starts with a supportive stance that explores patients’ strengths and sources of resilience – after all, the same cognitive, financial, and social capacities required to travel may also facilitate treatment. In our experience, travelers with paranoia or involuntary ED presentations are resistant to accessing care: emergency providers should strive to help these patients feel comfortable returning to the ED should new problems arise.

Tourists and travelers with reliable plans to return to usual care in another city may be assisted with bridging prescriptions or guidance to urgent care providers for help until returning to a usual provider (as with Case 2). Absent concerning side effects, changes in psychiatric medications and dosing are best handled by the patient’s regular provider rather than in an acute ED visit.

### “Bus therapy”

Some hospitals provide a one-way bus ticket to another state for patients with challenging psychiatric presentation and recurrent service utilization.^34^,^35^ This practice is pejoratively called “Greyhound Therapy.”^34^ There are no data as to the outcomes of bus therapy. In some instances, travelers specifically request help with a bus ticket, and clinicians are able to speak with family members or outpatient providers in the city to which patients wish to return. Under these circumstances it is reasonable to facilitate travel in hopes of helping the patient re-establish contact with an established social and healthcare network.

However, providing a bus ticket to a patient with no plan for shelter or resources in the destination city is “patient dumping.”^35^ Bus therapy in this fashion is unethical and discouraged. Dumping constitutes a failure of the duty to care for patients and distorts the just distribution of mental health resources.

## FUTURE DIRECTIONS

Much about patient peregrination remains to be discovered. The frequency and costs associated with peregrination are unknown, as are firm data regarding psychiatric and medical morbidity among these patients. Studying these patients is difficult: Travelers move across regions and healthcare systems, change insurance providers frequently, and are unlikely to participate in longitudinal studies. Widespread adoption of electronic medical records may facilitate an investigation of these patients. At a local level, hospitals can develop case definitions and collect information on the care needs of travelers. It is unclear what outcomes would be of value in evaluating the care of traveling patients.

## CONCLUSION

Peregrinating psychiatric patients are commonly seen in the ED. These patients consume significant attention and resources in the emergency department. The historical association of traveling with factitious disorder has obscured the broad range of diagnoses associated with traveling patients (Table 2). Indeed, clinical practice and limited data suggest that travelers represent a patient population with significant psychopathology. Emergency clinicians’ assessment and treatment must reflect the unique needs of the peregrinating patient.

## Figures and Tables

**Figure:**
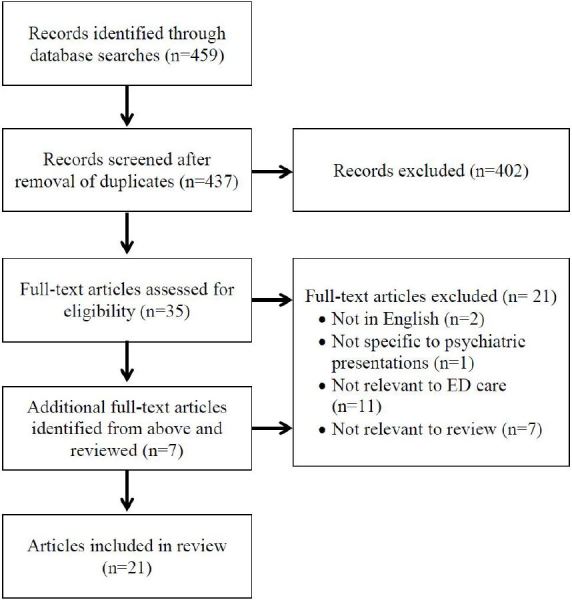
Article selection flow chart. *ED,* emergency department

**Table 1:** Key questions for evaluating the peregrinating emergency department patient.

Question	Clinical information
How long have you been in this city?	Collect history of present illness and consider time course of symptoms.
What brought you to this city?	Understand motivation.
Where are you staying? Where do you eat? What is your income source?	Assess for grave disability and identify basic needs.
How often do you move?	Learn pattern of behavior and usual coping styles.
Who do you know here? Any friends? Family?	Identify sources of support.
How long do you plan to stay here?	Begin planning for follow-up care.

**Table 2:** Common diagnoses among peregrinating psychiatric patients.

Anxiety, depression, or crisis after re-location for work or personal reasons
Schizophrenia
Bipolar mania
Delusional disorder
Substance abuse
Personality disorders, including borderline and antisocial
Factitious disorder
Dissociative disorders
Malingering
Delirium, cognitive impairment, or psychosis secondary to jet travel
